# The Urgent Threat of *Clostridioides difficile* Infection: A Glimpse of the Drugs of the Future, with Related Patents and Prospects

**DOI:** 10.3390/biomedicines11020426

**Published:** 2023-02-01

**Authors:** Ahmed S. Alshrari, Shuaibu Abdullahi Hudu, Fayig Elmigdadi, Mohd. Imran

**Affiliations:** 1 Department of Medical Laboratory Technology, College of Applied Medical Sciences, Northern Border University, Arar 91431, Saudi Arabia; 2 Department of Basic Medical and Dental Sciences, Faculty of Dentistry, Zarqa University, Zarqa 13110, Jordan; 3 Department of Pharmaceutical Chemistry, Faculty of Pharmacy, Northern Border University, Rafha 91911, Saudi Arabia

**Keywords:** *Clostridioides difficile*, urgent threat, drugs, discovery, development, patent

## Abstract

*Clostridioides difficile* infection (CDI) is an urgent threat and unmet medical need. The current treatments for CDI are not enough to fight the burden of CDI and recurrent CDI (r-CDI). This review aims to highlight the future drugs for CDI and their related patented applications. The non-patent literature was collected from PubMed and various authentic websites of pharmaceutical industries. The patent literature was collected from free patent databases. Many possible drugs of the future for CDI, with diverse mechanisms of action, are in development in the form of microbiota-modulating agents (e.g., ADS024, CP101, RBX2660, RBX7455, SYN-004, SER-109, VE303, DAV132, MET-2, and BB128), small molecules (e.g., ridinilazole, ibezapolstat, CRS3123, DNV3837, MGB-BP-3, alanyl-L-glutamine, and TNP-2198), antibodies (e.g., IM-01 and LMN-201), and non-toxic strains of CD (e.g., NTCD-M3). The development of some therapeutic agents (e.g., DS-2969b, OPS-2071, cadazolid, misoprostol, ramoplanin, KB109, LFF571, and Ramizol) stopped due to failed clinical trials or unknown reasons. The patent literature reveals some important inventions for the existing treatments of CDI and supports the possibility of developing more and better CDI-treatment-based inventions, including patient-compliant dosage forms, targeted drug delivery, drug combinations of anti-CDI drugs possessing diverse mechanisms of action, probiotic and enzymatic supplements, and vaccines. The current pipeline of anti-CDI medications appears promising. However, it will be fascinating to see how many of the cited are successful in gaining approval from drug regulators such as the US FDA and becoming medicines for CDI and r-CDI.

## 1. Introduction

*Clostridioides difficile* (CD) (formerly *Clostridium difficile*) is a pore-producing, anaerobic, motile, rod-shaped, pleomorphic, ubiquitous, soil-prevalent, and contagious Gram-positive bacterium [1]. CD infection (CDI) can be acquired through physical contact with CD-contaminated persons, animals, soil, and other items, and this is because CD is very difficult to remove from the surface of a person or object [2,3]. This bacterium can enter the body when a contaminated hand or items come into contact with the body’s mucous membranes, such as the mouth or eyes. Upon entering the body, CD produces enterotoxin A and enterotoxin B, which are responsible for diarrhea and inflammatory conditions such as colitis [4]. Some CD strains (027/BI/NAP1) produce a third toxin (binary toxin) and cause a more severe form of CDI [5]. Untreated CDI is fatal and may also lead to colon cancer [6]. CD produces heat-resistant spores under stressful conditions that are capable of tolerating extreme environments. The spores of CD can survive in a patient’s body for a long time and significantly increase the chances of recurrence (one in every six patients) of CDI (r-CDI) [7]. The risk factors for developing CDI include old age, history of CDI, recent hospital visit/stay, use of certain drugs (e.g., proton-pump inhibitors, diuretics, etc.), type of food, and compromised immunity (e.g., patients with AIDS, cancer, organ transplants, etc.) [8]. The irrational use of antibiotics (e.g., amoxicillin, ceftriaxone, cephalexin, clindamycin, levofloxacin, meropenem, and the combinations of piperacillin plus tazobactam) disrupts the healthy microbiome by wiping out the beneficial and the pathogenic bacteria [9,10]. Accordingly, people on antibiotic therapy are 7–10 times more susceptible to developing CDI [9]. Common CDI symptoms comprise diarrhea, leukocytosis, fever, stomach pain, nausea, and loss of appetite [9]. The severe manifestations comprise septic shock, toxic megacolon, perforated intestine, and death [9,11]. CDI can be diagnosed by enzyme immunoassay, nucleic acid amplification testing, colonic histopathology, and testing for enterotoxin A and enterotoxin B [12,13]. However, there is no optimal single test for the proper diagnosis of CDI [13]. CDI has a substantial recurrence rate and can occur in episodes [7]. The first episode of CDI is treatable with fidaxomicin (all forms of CDI), nitazoxanide (all forms of CDI), rifaximin (all forms of CDI), metronidazole (mild CDI), vancomycin (mild-to-severe CDI), tigecycline (refractory CDI), and colonic surgery [7,14,15]. Cases of r-CDI can be treated with fidaxomicin, vancomycin, rifaximin, immunoglobulin, monoclonal antibodies (e.g., bezlotoxumab), and transplantation of the fecal microbiota [14,15]. The literature further suggests more evidence-based efficacious treatments (e.g., bacitracin, fusidic acid, teicoplanin, rifampicin, and tolevamer) with unclear roles [14].

**Figure 1:**
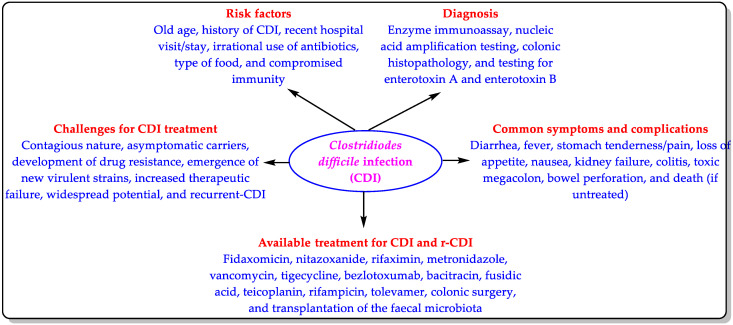
Risk factors, symptoms, diagnosis, treatment, and challenges of CDI.

CDI is an antimicrobial-resistance-derived infection that occurs annually in thousands of people. According to the Centers for Disease Control and Prevention (CDC) report of 2019, 223,900 CD-infected patients were hospitalized, 12,800 patients died, and CDI was responsible for about USD 1 billion in healthcare costs in 2017 [9]. It is important to note that this CDC report did not consider the non-hospitalized cases of CDI. This means that the number of CDI cases must have been higher than indicated in this report. CD is rarely resistant to antibiotics, but the development of drug resistance and the emergence of CD virulent strains are of great concern [9,16]. Due to increased therapeutic failure over the past few years, CD is now seen as a hazard to public health [17]. Furthermore, CD has wide transmission potential. Therefore, CDI is classified as an urgent threat and one of the CDC’s top five “priority dangers” [9] (Figure 1).

Alternative tactics are essential to combat CDI, including the discovery of new drugs and treatments for CDI. Some reviews and reports have discussed the development of new drugs and better treatments for CDI [10,18,19,20,21,22,23,24,25]. However, these reviews are silent about the patent literature of the developing/existing treatments for CDI, and they also overlook some developing drugs in their text. This article highlights the important foreseeable future drugs for CDI, along with their patent literature. This article will be of benefit to scientists developing innovative and patentable treatments for CDI.

## 2. Methodology

The updated non-patent literature search for this review was carried out on 12 December 2022, utilizing the keyword “difficile” in the title/abstract section of PubMed. The results were subsequently filtered using the “Review”, “clinical trial”, and “randomized controlled trial” filters of PubMed. The important and foreseeable drugs of the future for CDI were identified based on this PubMed search and are discussed in the review. The patent literature search was also performed on free patent databases (USPTO, Patentscope, and Espacenet) [26,27,28] on 12 December 2022, utilizing the names of drugs identified in the PubMed search. The patents explicitly claiming the use of drugs for treating CDI were included in the review. This patent search cannot be thought of as a complete search. However, the cited patents/patent applications in the manuscripts are sufficient to provide insight into the current and foreseeable inventions for CDI drugs. The readers can perform structure searches and CAS number searches for the new drugs utilizing the paid SciFinder database for further information about the specific drugs. The clinical trial database was used to identify the clinical study data of the drugs cited in the manuscript [29]. Detailed information about the National Clinical Trial (NCT) numbers cited in the text can be obtained from the clinical trial database by searching the NCT numbers in the database [29]. The chemical information of the drugs was taken from the PubChem website, along with the data available from the respective pharmaceutical industries [30].

## 3. Existing Treatments for CDI and Related Patents

The existing treatments for CDI have been discussed in the literature [7,14,15], and they are also mentioned in Figure 1. Accordingly, we included some important patents/patent applications for the existing treatments (Table 1).

## 4. Drugs of the Future for CDI

### 4.1. Microbiome-Modulating Agents (MMA)

**Figure 2:**
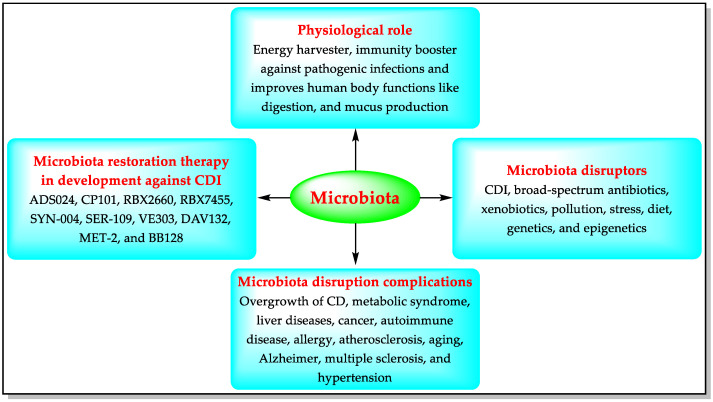
Functions of the microbiota, its disruptors, and microbiota-based CDI therapies in development.

More than a thousand harmless bacterial species are present in the human digestive system, forming a community of germs known as the “microbiota”. The healthy microbiota provides immunity against pathogenic infections and helps the human body to function better [50,51]. Imbalance in the natural microbiota is called dysbiosis (losing good bacteria, or excessive growth of harmful bacteria such as CD). Microbiome disruptors can trigger dysbiosis, including pathogenic infections such as CDI [51]. A decrease in the colonization of some microbes (e.g., Bacteroidetes, Firmicutes, Faecalibacterium, and Bifidobacterium) has been observed in CD-infected patients [50,51]. Untreated dysbiosis can lead to complications such as stomach cancer [51]. Modulation of the gut microbiota is a strategy aiming at reversing dysbiosis by using microbiome-modulating agents (MMAs) such as prebiotics, probiotics, postbiotics, and transplanting fecal microbiota. The microbiome-modulating agents modify the microbiota by eliminating or preventing the transport of microbiome disruptors, including CD [50,51]. CD’s growth increases in disrupted microbiota, producing toxins and causing CDI’s pathologies, including diarrhea, colitis, and toxic megacolon. The MMAs preserve the endogenous gastrointestinal microbiota and help to prevent/treat CDI and r-CDI. Accordingly, pharmaceutical companies are developing MMAs for microbiota restoration therapy against CDI [50,51] (Figure 2).

#### 4.1.1. ADS024 (ART24)

ADS024, formerly ART24, is a new direct-acting live biotherapeutic (a strain of *Bacillus velezensis*). Adiso Therapeutics is developing ADS024 as a potential candidate for preventing the recurrence of CDI [52]. ADS024 kills CD directly by inhibiting its translation and permeabilization processes. ADS024 also produces proteases that break down the CD toxins responsible for most of the symptoms of CDI. Phase I of the clinical trial (NCT04891965) of the capsule containing lyophilized ADS024 was completed on October 13, 2022. The United States Food and Drug Administration has granted fast-track designation to ADS024 [53].

**US11419900B2** (Artugen Therapeutics) claims a composition comprising lyophilized spores of ART24 (NCIMB Accession No. 43088) and a preservative (i.e., sucrose, trehalose, sodium ascorbate, and glutathione) for treating CDI [54].

**WO2021116983A1** (Artugen Therapeutics) claims an edible product (dietary supplement) comprising lyophilized spores or a vegetative form of ART24, along with other edible products (e.g., fermented food products, soybeans, mushrooms, mung beans, locust beans, rice, or extracts thereof). It also claims a method of treating CDI using the proposed edible product [55].

#### 4.1.2. CP101

Finch Therapeutics is developing P101 as an oral investigational microbiome drug (full-spectrum microbiota containing diverse microorganisms) to prevent recurring CDI. P101 has entered a clinical phase III trial (NCT05153499) based on the extremely positive results of its clinical phase II trial (NCT03497806). The US FDA has given breakthrough and fast-track designation to P101. The lyophilized P101 capsules are designed to release the drug at the local site of the CDI. The action of CP101 is to restore the diversity of the microbiome, addressing the disruption that can lead to r-CDI [56,57,58]. The website of Finch Therapeutics lists many patents on pharmaceutical compositions for fecal floral transplantation. However, the authors could not properly identify the patents related to CP101 [59].

#### 4.1.3. RBX2660

Rebiotix is developing the biotherapeutic RBX2660 (a gut-microbiome-based enema formulation made from prescreened human donor stool containing a diverse set of microorganisms) to treat CDI [60,61,62]. One clinical trial for an RBX2660-based microbiota restoration therapy (phase III, PUNCH-CD3, enema suspension formulation, NCT03244644) has been completed, while another clinical phase III trial (PUNCH CD3-OLS, enema formulation, NCT03931941) is underway [60,61]. RBX2660 has received important designations (fast-track, breakthrough, and orphan drugs) from the US FDA [60,61].

**US9782445B2** (Rebiotix) claims a method of treating CDI using a microbiota restoration therapy composition containing specified components (i.e., human fecal sample, polyethylene glycol, and saline) [63]. The specification of **US9782445B2** discusses the clinical effects of RBX2660 (microbiota suspension as an enema) on CD-infected patients [63].

#### 4.1.4. RBX7455

Rebiotix is also developing the capsule formulation of another live biotherapeutic—RBX7455 (a room-temperature-stable, lyophilized, non-frozen, orally delivered microbiota-restoring investigational drug containing a diverse set of microorganisms)—for CDI [64,65]. The clinical phase I trial (NCT02981316) of RBX7455 has been completed [64,65]. Apart from CDI, RBX7455 is also being investigated for hepatic encephalopathy (phase II, NCT04155099), breast cancer (phase I, NCT04139993), and Crohn’s disease (phase I, NCT03378167). 

**WO2022051610A1** (Ferring) claims a method for treating r-CDI using a capsule formulation containing a lyophilized material comprising fecal microbiota, polyethylene glycol, trehalose, sucrose, and glycerin [66]. The specification of **WO2022051610A1** also discusses the phase I clinical trial of RBX7455 [66].

#### 4.1.5. SYN-004 (Ribaxamase)

Theriva Biologics is developing ribaxamase (an oral beta-lactamase enzyme) to be taken along with intravenous beta-lactam antibiotics to break down excess medications in the upper gastrointestinal tract before they disturb the gut microbiota and cause CDI [67,68]. SYN-004 is anticipated to shield the gut microbiota from change, reducing harmful consequences such as CDI, opportunistic pathogen colonization, and the development of resistance to antibiotics in the gut microbiome [68]. A phase II clinical trial (NCT02563106) related to the use of SYN-004 to prevent CDI in patients with lower respiratory tract infections has been completed. Another related clinical trial (phase I, NCT04692181) aiming to evaluate the tolerability and safety of oral SYN-004 is in progress.

**US2022218800A1** (Synthetic Biologics) claims a method for reducing the incidence and severity of dysbiosis associated with administering antibiotics using an effective amount of a beta-lactamase agent. This US patent application provides the nucleotide sequence of SYN-004 and exemplifies its dysbiosis prevention effects in different examples [69]. 

**US2019275120A1** (Synthetic Biologics) claims a method for treating or preventing infection (CDI) in patients determined to be resistant to an antibiotic, consisting of administering an effective amount of a beta-lactamase before or concurrently with the antibiotic or a different antibiotic [70].

#### 4.1.6. SER-109

Seres Therapeutics is developing SER-109 (live purified spores of Firmicutes) as a first-in-class groundbreaking oral investigational microbiome therapy for r-CDI [18,71]. The US FDA has granted SER-109 breakthrough therapy and orphan drug designation [18,71]. Two clinical phase III studies (NCT03183128 and NCT03183141) related to the effectiveness of SER-109 against r-CDI have been completed, whereas one expanded access study (NCT02437500) is in progress. Our patent search revealed some patents/patent applications assigned to Seres Therapeutics. However, none of them explicitly claimed SER-109 or an SER-109-based composition. Therefore, the authors have not incorporated patent information about SER-109. 

#### 4.1.7. VE303

Vedanta Biosciences is developing an oral capsule of VE303 (a specified bacterial consortium treatment comprising eight specified bacterial strains) to stop r-CDI. The clinical phase II trial (NCT03788434) of VE303 for the prevention of r-CDI has been completed [72,73].

**US2022143108A1** (Vedanta Biosciences) claims a method for decreasing dysbiosis, restoring the microbiome, and increasing the recovery of a healthy microbiome in a subject utilizing a composition comprising one or more purified bacterial strains to increase the recovery of a healthy microbiome [74]. This US patent application details the pharmacokinetic and pharmacodynamic activity of VE303.

#### 4.1.8. DAV132

Da Volterra is developing oral DAV132—an activated-charcoal-based colon-specific antibiotic inactivator and adsorbent that retains the gut microbiota, reduces dysbiosis, and avoids problems such as CDI and the development of antibiotic-resistant bacteria [75,76]. DAV132 can be taken orally in combination with antibiotics and does not affect the pharmacokinetics of the co-administered antibiotics [77]. Unabsorbed antibiotics and their residues bind to DAV132 and are removed from the patients through feces, preventing antibiotic-induced alteration of the microbiota [77]. The safety- and efficacy-based clinical phase II trial (NCT03710694) against CD was completed in 2019. 

**US10105322B2** (Synthetic Biologics) does not specifically relate to DAV132 but mentions DAV132 as a charcoal-based binder [78]. 

#### 4.1.9. MET-2 (Microbial Ecosystem Therapeutic-2)

NuBiyota is developing various multistrain microbiome therapeutic composition candidates, including the oral biotherapeutic MET-2, to treat CDI [79]. MET-2 is a novel therapy method developed as a substitute for fecal transplantation for incapacitating recurring CDI [80]. MET-2 is neither a medicine nor a biologic; rather, it is made up of 40 living bacteria that would usually be found in the human digestive tract of a person who is healthy [81]. The MET-2 based clinical phase I trial (NCT02865616) for treating CDI was completed in 2020.

**US2021069262A1** (NuBiyota) describes Microbial Ecosystem Therapeutic 2 (designated MET-2), including subgroups of MET-2 (e.g., MET-2A and MET-2B), different MET-2 strains, and their properties. It claims a method for treating dysbiosis (CDI) with MET-2 [82].

#### 4.1.10. BB128

BiomeBank is developing BB128, a live biotherapeutic comprising a lyophilized fecal microbiota. Its marketing authorization application has been submitted to the Therapeutic Goods Administration of Australia [26].

### 4.2. Small Molecules

**Figure 3:**
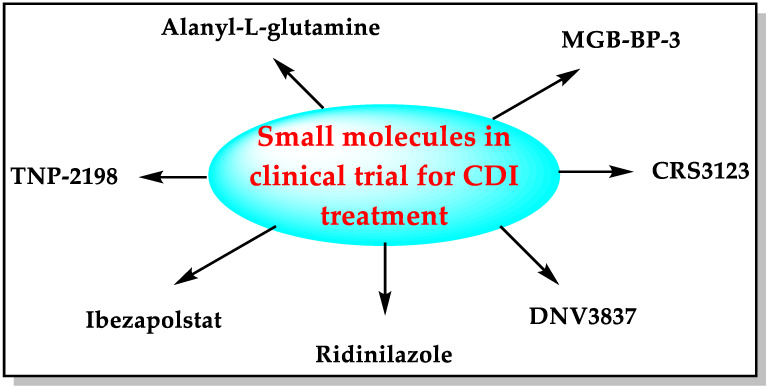
Small molecules in development for CDI treatment.

Small molecules such as fidaxomicin, nitazoxanide, rifaximin, metronidazole, vancomycin, and tigecycline are used in clinical practice to treat CDI [7,14,15]. The pharmaceutical industry is developing some new small molecules with diverse mechanisms of action to combat the urgent threat of CD (Figure 3). 

#### 4.2.1. Ridinilazole (SMT19969)

**Figure 4:**
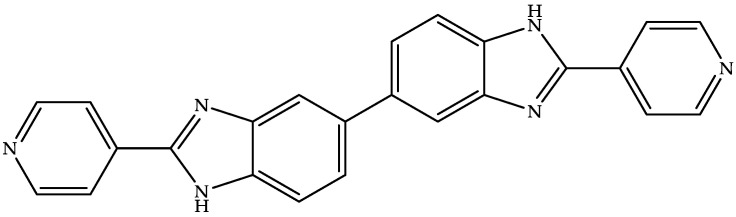
Chemical structure of ridinilazole.

Summit Therapeutics is developing an oral dosage form (aqueous suspension, hard gelatin capsule, and tablet) of ridinilazole (Figure 4)—a benzimidazole–pyridine derivative—to treat CDI [83,84].

Ridinilazole prevents cell division, reduces toxin production, and specifically kills CD, but it is harmless to the gut microbiota [84]. Its narrow spectrum, poor systemic absorption due to low solubility (BCS class IV), local action (lower intestine), and potency against CD make ridinilazole a desirable candidate for CDI treatment, with a low minimum inhibitory concentration (MIC) ranging from 3 to 25 µg/mL [84,85]. The US FDA has given fast-track, breakthrough, and qualified infectious disease product (QIPD) designation to ridinilazole [83]. Summit Therapeutics has completed two clinical phase III studies (NCT03595553 and NCT03595566) related to the comparison of ridinilazole and vancomycin for the treatment of CDI. Ridinilazole exhibits enhanced preservation of the human intestinal microbiota compared to vancomycin. In contrast, the third phase III study on the pharmacokinetics and tolerability of ridinilazole in adolescent subjects has been terminated.

**US2022226249A1** (Summit Therapeutics) claims a tablet formulation comprising ridinilazole crystal agglomerates and an intragranular solid phase incorporated in an extragranular solid phase [86]. This US patent application provides different crystal forms of ridinilazole and their preparation methods. It further mentions the advantages of the proposed tablet formulation over the aqueous suspension and immediate-release liquid-filled hard gelatin capsule. 

#### 4.2.2. Ibezapolstat (ACX-362E)

**Figure 5:**
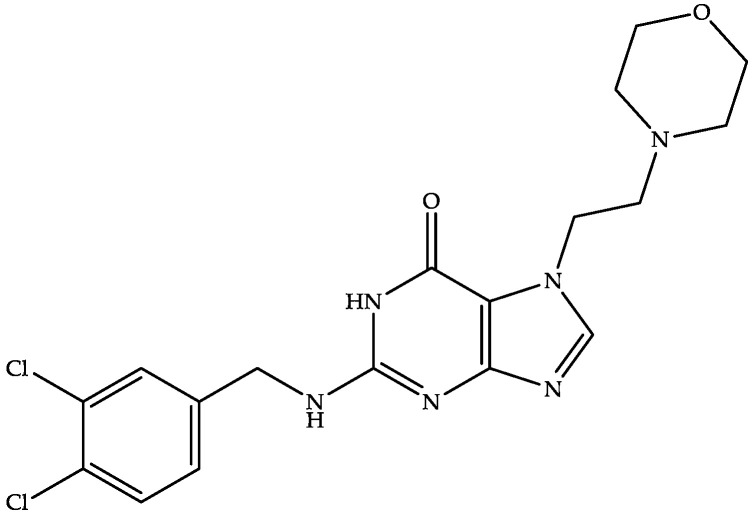
Chemical structure of ibezapolstat.

Acurx Pharmaceuticals is developing oral ibezapolstat (Figure 5)—a dichlorobenzyl purine derivative—as the first-in-class inhibitor of DNA polymerase IIIC (the enzyme responsible for the replication of bacterial cells) for the treatment of CDI [87,88]. Ibezapolstat has also been demonstrated to have an MIC between 1 and 8 µg/mL [88] and was active against CD in the clinical phase III trial (NCT04247542) [87,88].

The US FDA has also granted QIDP and fast-track status to ibezapolstat [87]. In addition to being effective against CDI, ibezapolstat also helps to reestablish a healthy microbiota in the intestinal tract. This dual property of ibezapolstat can make it the first-line therapy for CDI [87,88]. 

**US2022024925A1** (Acurx Pharmaceuticals) provides a short description of ibezapolstat. This patent application claims that deuterated analogs of ibezapolstat possess longer half-lives without affecting the compound’s bioavailability or safety [89].

#### 4.2.3. CRS3123

**Figure 6:**
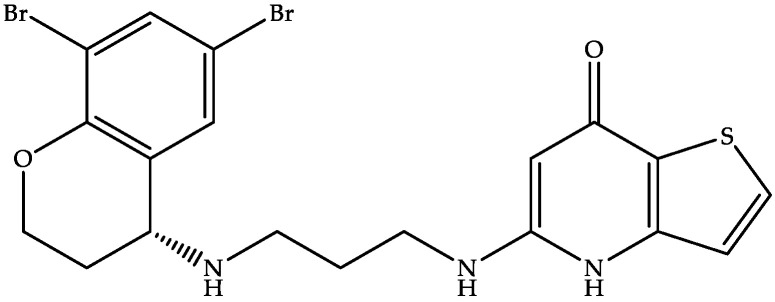
Chemical structure of CRS3123.

CRS3123, a benzopyran derivative (Figure 6), is a methionyl-tRNA synthase inhibitor capable of inhibiting CD spores’ protein translation [90,91].

Crestone Pharma is developing an oral dosage form of CRS3123 for CDI. CRS3123 has demonstrated high potency against all clinical isolates of CD, inhibits toxin production in CD, rapidly relieves symptoms of CDI, inhibits sporulation, and has a novel mechanism of action with an MIC value of 1 µg/mL and a mutant concentration value ranging from 16 to 128 µg/mL [91]. CRS3123 has completed phase I clinical trials (NCT01551004 and NCT02106338) and is currently in a phase II clinical trial (NCT04781387) for treating CDI [90,91].

**US2022000884A1** (Vanderbilt University) claims a method for treating or preventing the recurrence of CDI using a combination of misoprostol and CRS3123 [92]. However, no example has been provided in the specification for the activity of this combination against CDI.

#### 4.2.4. DNV3837

**Figure 7:**
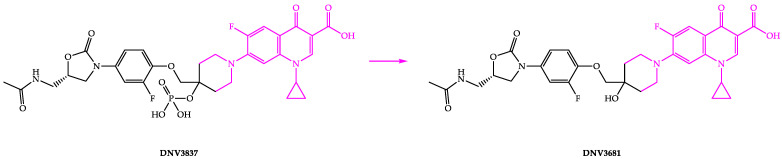
Chemical structures of DNV3837 and DNV3681.

DNV3837 is a quinolone-based oxazolidinone hybrid compound with a dual mechanism of action (Figure 7). DNV3837 inhibits bacterial DNA gyrase due to its fluoroquinolone moiety and protein synthesis due to its oxazolidinone moiety, and it has been demonstrated to have an MIC of 0.25 µg/mL [93]. Blood esterases dephosphorylate DNV3837 (a water-soluble prodrug) after intravenous administration to provide the active form of the drug (DNV3681) [22,94,95]. DNV3837 is the first parenteral medicine that targets the intestine and generates high exposure of the active ingredient DNV3681 in the intestine’s tissue.

Deinove is developing intravenous DNV3837 for treating severe CDI and is in a phase II clinical trial (NCT03988855). The US FDA has granted fast-track and qualified infectious disease product (QIDP) designation to DNV3837 to expedite its development [94]. Our patent search on free databases did not reveal any relevant patent/application for DNV3837 and DNV3681.

#### 4.2.5. MGB-BP-3

**Figure 8:**
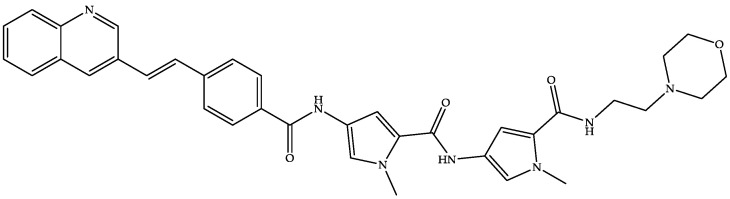
Chemical structure of MGB-BP-3.

MGB Biopharma is developing oral MGB-BP-3 (a quinoline–distamycin-based synthetic polyamide and genetic transcription inhibitor) (Figure 8) as a possible first-line treatment capable of providing a sustained cure for CDI [22,96,97].

As per the website of MGB Biopharma, MGB-BP-3 is in a clinical phase III trial for treating CDI after its successful completion of phase I (NCT02518607) and phase II (NCT03824795) trials [96]. MGB-BP-3 attaches to the tiny grooves in bacterial DNA formed by the closeness of the backbones of the DNA strands [22]. MGB-BP-3 has demonstrated a potent bactericidal effect against the NAP1/027 strain of CD, with a faster onset of action than vancomycin and an MIC of 0.25 μg/mL [22,96,98]. The US FDA has granted QIPD and fast-track status to MGB-BP-3 [22]. Our patent search on free databases revealed some patents on minor groove binders but did not reveal any hits for the term MGB-BP-3.

#### 4.2.6. Alanyl-L-glutamine (DB11876)

**Figure 9:**
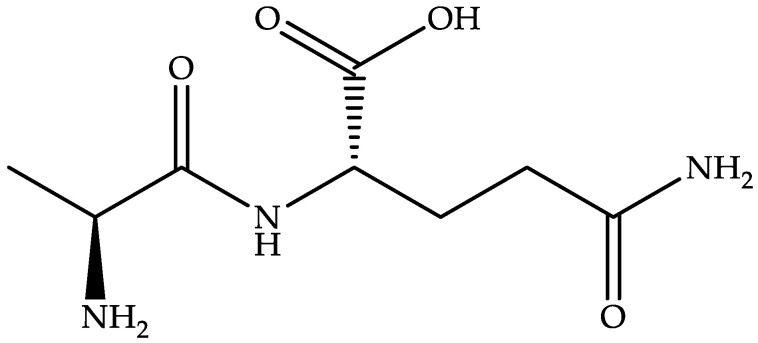
Chemical structure of alanyl-L-glutamine.

Alanyl-L-glutamine, a water-soluble L-alanine- and glutamine-based dipeptide (Figure 9), is a dietary supplement that improves electrolyte absorption and endurance during exercise [22].

Alanyl-L-glutamine blocks caspase-8 activation, suppresses T84-cell apoptosis, lessens the disruption and secretion of intestinal cells brought on by CD toxin A, and has demonstrated an MIC of 0.25 µg/mL [99]. An alanyl-L-glutamine supplementation-based phase II clinical trial (NCT02053350) for treating CDI has been terminated. Still, another interventional phase II clinical trial (NCT04305769) has been started by the University of Virginia in collaboration with Imperial College London. 

**CN103221420A** (University of Virginia) claims to use alanine–glutamine-rich heterologous polypeptides to treat CDI [100]. This patent application states alanine–glutamine as a stable derivative of glutamine that can be used not only for malnourished children suffering from diarrhea, but also for patients who receive parenteral fluid or tube feeding for too long or have damaged intestinal mucosa due to infection or chemotherapy.

#### 4.2.7. TNP-2198 (Rifasutenizol) 

TenNor Therapeutics is developing TNP-2198 as a stable multitarget rifamycin–nitroimidazole hybrid antimicrobial with a novel and synergistic mechanism of action for the treatment of anaerobic and microaerobic bacterial infections, including CD and *H. pylori*, with MIC values ranging from 0.002 to 4 µg/mL [75,101,102]. The rifamycin component of the rifasutenizol binds to RNA polymerase, whereas the whole nitroimidazole component directly interacts with the DNA template in the active center cleft of the RNA polymerase [75]. TNP-2198 is in a phase Ib/IIa clinical trial in China (CTR20190734) [102].

**CN104971061B** (TenNor Therapeutics) claims to use a rifamycin–nitroimidazole conjugate to prepare drugs against *H. pylori* and CDI [49].

**CN106822119A** (TenNor Therapeutics) claims to use a rifamycin–nitroimidazole conjugate to inhibit gastrointestinal anaerobic or facultative anaerobic production of bacteria comprising CD [103].

### 4.3. Miscellaneous Therapies against CDI

#### 4.3.1. IM-01

ImmuniMed is developing IM-01, an egg-derived orally effective polyclonal antibody, which is in a phase II clinical trial (NCT04121169) for the treatment of mild-to-moderate CDI [75,104,105]. IM-01 neutralizes the CD toxins and inhibits the growth of spores and vegetative forms of CD [105]. ImmuniMed lists certain strategic and competitive advantages (i.e., safety, efficacy, and scalability) of antibody therapy over other therapies [104]. Since IM-01 is derived from chicken eggs, which form part of the human diet, it is considered safe by the US FDA [104].

**US9873732B2** (ImmuniMed) claims a method for treating CDI by administering an effective amount of egg-derived polyclonal antibody, wherein the CD strain is selected from a group consisting of NAP/B1/027, CCL678, HMC553, Pitt45, CD196, Montreal 5, Montreal 7.1, MH5, Pitt2, CCL14137, UVA17, UVA30/TL42, and Pitt7 [106].

#### 4.3.2. LMN-201

Lumen Bioscience is developing LMN-201, the first-in-class orally bioavailable and highly potent therapeutic monoclonal antibody cocktail against CDI. LMN-201 comprises three antibody-like proteins (to neutralize CD toxins such as toxin B of CDI) and one lysozyme-like enzymatic protein (which destroys the cell wall of CD) [107,108]. This cocktail is believed to be more potent than single antibodies and the existing antibody treatments for CDI [108]. The interventional phase I clinical trial (NCT04893239) was completed in February 2022. A phase II/III clinical trial (NCT05330182) related to the prevention of r-CDI with LMN-201 has also been started [107,108]. The enteric-coated capsule of LMN-201 consists of a cocktail of proteins in the edible, dried, and non-viable biomass of spirulina (*Arthrospira platensis*) [107].

**US2021338751A1** (Lumen Bioscience) claims a non-parenterally delivered composition of recombinant spirulina with two or more proteins that bind to a Clostridium toxin (toxin B) and a lysine [109]. This was validated in a completed phase I clinical trial that demonstrated strong gastric protease stability, and it is currently in a phase II clinical trial (NCT05330182).

#### 4.3.3. NTCD-M3

NTCD-M3 (formerly VP 20621) is a natural, non-toxic strain of CD (NTCD) that lacks the genes responsible for expressing CD toxins [110,111,112]. Destiny Pharma is developing an oral formulation of the spores of NTCD-M3 that temporarily colonizes and makes a “ground cover” in the colon without producing any symptoms of CDI. This “ground cover” does not allow the proliferation of the pathogenic CD in the colon [110,112]. After a few weeks of treatment, the gut microbiota returns to normal after NTCD-M3 temporarily takes root in the intestinal mucosa, without producing any symptoms. According to Destiny Pharma, NTCD-M3 is in a phase III clinical trial [110]. 

**EP3871683A1** (Paris University) claims a non-toxicogenic strain of CD (ribotype CE032 and ribotype CE847) for use in the primary prevention and treatment of infections associated with CD. However, it is silent about NTCD-M3 or VP 20621 [113].

The summary of the novel treatments is summarized in Table 2.

## 5. Drugs Not in Active Development

The WHO’s report lists some anti-CD drugs that are not in active development or clinical trials [75]. A summary of these drugs is provided in Table 3.

## 6. Discussion

CDI, an urgent threat and unmet medical need, is a hospital-acquired and community-acquired CD-toxin-mediated enteric infection [106,125]. The current treatments for CDI have limited efficacy and are insufficient to combat this urgent threat (Figure 1). Accordingly, new therapeutics for CDI are urgently needed.

Many therapeutic agents, including MAAs (Figure 2), small molecules (Figure 3), antibodies (IM-01 and LMN-201), and non-toxic strains of CD (NTCD-M3), with diverse mechanisms of action as possible future drugs for CDI, are under development. These therapeutic agents have been developed utilizing different approaches, including the development of bacteria-based intestinal microbiota, CD toxin inactivators/degraders, antibiotic adsorbents/degraders, inhibitors of the CD-specific targets, preparation of hybrid molecules with two known antibacterial pharmacophores, drug repurposing, diet-derived antibodies, and combinations of proteins/antibodies. Microbiota disruption can cause many complications in addition to CDI (Figure 2). MAAs can help to avoid these complications. This feature of MAAs gives them an edge over other molecules/antibodies described in this manuscript. The development of some anti-CD drugs has been halted (e.g., DS-2969b, OPS-2071, cadazolid, misoprostol, ramoplanin, KB109, LFF571, and Ramizol) due to failed clinical trials or unknown reasons. These failures seem discouraging, but they cannot stop the scientific community from developing better treatments for CDI.

Developing innovations against urgent threats such as CDI is costly and challenging. Accordingly, protecting inventions via patent filings is one strategy adopted in the pharmaceutical industry [126,127,128]. The authors performed a patent search to identify different types of CDI-based treatments. Table 1 reflects the different inventions using the existing CDI treatments, comprising methods of treating CDI and its associated diarrhea, new uses of anti-CD drugs, different dosage forms (e.g., tablets, powders, suspensions, dosage regimens with improved efficacy, solid dispersions, nanoemulsions, controlled-release compositions, drug combinations, enteric-coated compositions, capsules, solutions, colon-specific drug delivery, and injections), anti-CD polypeptides, and hybrid molecules. Other important inventions related to the cited drugs of the future have also been identified—for example, lyophilized drugs [54], edible products containing anti-CD drugs [55], methods for decreasing dysbiosis with the drug [74], charcoal-based binders [78], microbial ecosystem therapeutics [82], polymorphs [86], deuterated analogs [89], treating r-CDI with drug combinations [92], delivery methods with edible products [109], and non-toxicogenic strains of CD [113]. All of these inventions are useful to scientists developing new and better treatments for CDI and r-CDI.

The most common precipitant of CDI is the irrational antibiotic use that is leading to an increase in the prevalence of antimicrobial resistance. The drug-resistant strains of CD are also increasing [86]. Therefore, controlling antimicrobial resistance and developing novel therapies for drug-resistant strains of CD may prove to be an effective strategy against CDI. Moreover, the development of new treatments seems insufficient to fight the drug-resistant CD strains. Treatment of drug-resistant CD strains is possible with proper and timely diagnosis to avoid therapeutic failure. A parallel development for the diagnostic methods is also warranted [129]. The prevention of the sporulation of CD is important to fight r-CDI. Ridinilazole, CRS3123, and ramoplanin encompass this property. However, more research is needed to develop anti-CD drugs that are active against the sporulation of CD.

The toxins of CD (toxin A and toxin B) are responsible for the inflammation, degradation, and damage to the intestinal epithelial cells. Fidaxomicin inhibits CD toxin (A and B) synthesis and spore formation, with a low rate of r-CDI. However, fidaxomicin is expensive and has some side effects (e.g., fever, dizziness, and increased levels of hepatic enzymes) [130]. Developing more antidotes and antibodies for CD toxins will effectively combat CDI’s pathogenesis [131].

The drug repurposing approach is a cheaper and faster way to discover new drugs for use against a disease. Some anti-CD drugs are in development using this strategy. There are a lot of safe and effective antimicrobials and food supplements that can be assessed to ensure their anti-CDI potential. Accordingly, the drug repurposing approach to discover safe and effective anti-CD molecules seems promising [100,106]. 

The development of anti-CD vaccines is one of the areas in the fight against CDI and r-CDI. A live, genetically engineered, attenuated Salmonella-vector-based vaccine comprising CD antigens has been reported [132]. A vaccine containing degenerative toxins A and B is being studied to prevent r-CDI, but its commercialization is expected to take considerable time [130]. Furthermore, the multilocus sequence typing (MLST) database reports two clades of CD: Clade I and Clade II. Clade I of CD is the most common and diverse form of CDI (accounting for about 64% of CDI) [129]. Research investigating the two different clades of CD is also advocated.

Most CDI treatments are administered orally. The oral dosage form may be non-compliant with geriatric and pediatric patients. Accordingly, developing suppositories, enemas, and targeted delivery systems that allow for effective distribution and improved retention of the therapeutic agent or agents in the affected area will benefit the patients [100,133].

An ideal drug for CDI should have low oral bioavailability, be effective against all CD strains, prevent sporulation and germination, have little effect on the gut microbiota, have a narrow spectrum, demonstrate the fewest drug interactions (e.g., food, drug, and disease interactions), and be patient-compliant for all patient populations (e.g., adult, pediatric, geriatric, pregnant women, etc.) [125]. However, all of these properties may not be present in a single therapeutic agent. Accordingly, the development of combinations of anti-CD drugs possessing different mechanisms of action (e.g., CD killer/inhibitors, CD toxin inactivators/degraders, microbiota, probiotics, antibiotic adsorbents/degraders, etc.) may provide a better treatment option for CDI and r-CDI. The development of novel and cost-effective dosage regimens of anti-CDI drugs also needs to be investigated. Supplementing digestive enzymes and probiotics may also provide a reliable therapy for CDI [55]. The binary toxin-producing strains of CD are among the main causes of r-CDI [5]. There are some drugs (e.g., LMN-201, IM-01, and fidaxomicin) that neutralize CD toxins. However, the data are unclear about their efficacy against the binary toxin produced by CD. This fact necessitates the development of drugs that can neutralize binary toxins of CD. 

## 7. Conclusions

Many possible future drugs (e.g., microbiota, small molecules, antibodies, non-toxic strains of CD, etc.) for the urgent treatment of CDI and r-CDI, with various mechanisms of action, are under development. These drugs have been invented utilizing different approaches to drug discovery and development. However, it will be interesting to see how many cited molecules receive approval from drug authorities such as the US FDA and become successful treatments for CDI and r-CDI. The authors also foresee a great scope in developing anti-CDI vaccines, drug combinations, novel treatment regimens, and targeted drug delivery systems as innovative treatments for CDI and r-CDI.

## Figures and Tables

**Table 1:** Important and relevant inventions using the available treatments for CDI.

Patent/Application Number (Applicant)	Summary
Fidaxomicin	
**US9808530B2** (Astellas Pharma)	A pharmaceutical composition (a dry powder for an aqueous suspension, a dry granulate for an aqueous suspension, or a dispersible tablet for an aqueous suspension) to treat CDI comprising fidaxomicin and xanthan gum as an excipient, wherein the excipient is present in an amount to prevent foaming of the tiacumicin B compound in water [31].
**US10548912B2** (Astellas Pharma)	A patient-compliant novel dosage regimen of fidaxomicin (initial course, monitoring, assessment, and drug switching for specified days) with improved efficacy for CDI, r-CDI, and CD toxins [32]. The proposed regimen is also supposed to reduce the chances of r-CDI to <5% and be cost-effective.
**WO2014135891A1** (Cipla Limited)	A pharmaceutical composition for rectal administration in the form of a foam comprising fidaxomicin and a suitable pharmaceutically acceptable excipient (e.g., propellants, emollient, humectants, pH-adjusting agent, surfactants, foaming agents, antioxidants, lubricants, etc.) for the treatment or maintenance of remission of infections caused by CD. The advantages of the claimed invention include targeted delivery, treatment of a large surface area, and bypassing the first-pass metabolism [33].
**Nitazoxanide**	
**CN114044761B** (Chengdu Biobel Biotechnology)	Nitazoxanide-based small molecules, which can effectively inhibit CD growth, have lower toxicity than nitazoxanide, and have a low impact on the intestinal flora [34].
**Rifaximin**	
**US11224591B2** (Cipla)	A pharmaceutical composition of rifaximin for CDI comprising at least one bioavailability-enhancing agent (piperine). The experiments of this patent demonstrate a relative bioavailability of 189% when rifaximin is administered with piperine [35].
**US8383151B2** (Lupin)	A more patient-compliant controlled-release pharmaceutical composition of rifaximin (administered once a day) than Xifaxan (200 mg tablet of rifaximin, administered three times a day) to treat CDI [36]. The experimental results also establish similar efficacy of Xifaxan and the claimed controlled-release composition of rifaximin.
**US9452157B2** (Alfa Wassermann)	A pharmaceutical composition for CDI comprising rifaximin and an amino acid (e.g., histidine, phenylalanine, proline, valine, leucine, etc.). The amino acids synergize the effect of rifaximin by allowing the release of higher rifaximin concentrations in the intestinal tract [37].
**WO2021058656A1** (Bausch Health Ireland)	A stable pharmaceutical composition with improved availability of rifaximin to the stomach and intestine, comprising rifaximin, castor oil, and a solubilizing excipient for treating CDI [38].
**Metronidazole**	
**US2017143707A1** (Kamtek)	A method of treating CDI with a synergistic combination of clofazimine, wherein the administration of said combination is synergistic compared with the administration of either clofazimine or metronidazole alone [39].
**Vancomycin**	
**US2019000789A1** (Universiteit Leiden)	A method of treating CDI with a combination of vancomycin and D-alanine amino acid. This patent application states that D-alanine lowers the vancomycin resistance of a vancomycin-resistant microorganism and helps improve and prolong the effect of vancomycin [40].
**EP0456418B1** (Kabushiki Kaisha Miyarisan Seibutsu Igaku Kenkyusho)	A composition comprising cells or spores of a butyric acid bacterium MIYAIRI 588 (CbM) and vancomycin to treat CDI. The short-term therapy with the claimed invention effectively prevented germination and propagation of CD in the intestine without impairing the intestinal microflora, thereby providing a complete cure for the disease, including preventing the recurrence of the disease [41].
**Tigecycline**	
**WO2007075794A2** (Wyeth Corporation)	Use of enteric-coated tigecycline for treating CDI-associated colitis. The enteric coating reduced the blood bioavailability of tigecycline and kept tigecycline within the stomach for local action against CD [42].
**Immunoglobulin**	
**US2018100008A1** (Vhsquared)	A polypeptide-based immunoglobulin that binds and neutralizes CD toxin B [43].
**US2018100009A1** (Vhsquared)	A polypeptide-based immunoglobulin that binds and neutralizes CD toxin A [44].
**Bezlotoxumab**	
**WO2018009789A1** (Vanderbilt University)	A method for treating or preventing CDI with a combination of misoprostol and bezlotoxumab. However, this patent application is silent about the experimental details of the claimed invention [45].
**CN106511266A** (Dong Shuqing)	An injection dosage form of bezlotoxumab to treat CDI [46].
**Bacitracin**	
**US5773000A** (Galagen)	A method of CDI with a combination of anti-CD bovine immunoglobulin, vancomycin, bacitracin, or metronidazole [47].
**Fusidic acid**	
**CA2767614C** (Cempra Pharmaceuticals)	A method of treating various bacterial infections, including CDI, with a loading dose (1200 mg) and maintenance dose (600 mg) of fusidic acid [48]. However, this patent is silent about the experimental details of the claimed invention against CD.
**Rifampicin**	
**CN104971061B** (Dannuo Pharmaceutical)	The use of a rifamycin–nitroimidazole coupling molecule to treat CDI, *H. pylori* tuberculosis, etc. [49].

**Table 2:** Summary of the novel treatments for CDI.

Drug (USFDA Designation)	Developer	Mechanism of Action	Phase
**ADS024** (Fast-track)	Adiso Therapeutics	MAA	Phase I completed
**CP101** (Breakthrough and fast-track)	Finch Therapeutics	MAA	Phase III
**RBX2660** (Fast-track, breakthrough, and orphan drug)	Rebiotix	MAA	Phase III
**RBX7455**	Rebiotix	MAA	Phase I completed
**SYN-004**	Theriva Biologics	MAA	Phase II completed
**SER-109** (Breakthrough and orphan drug)	Seres Therapeutics	MAA	Phase III completed
**VE303**	Vedanta Biosciences	MAA	Phase II completed
**DAV132**	Da Volterra	MAA	Phase II completed
**MET-2**	NuBiyota	MAA	Phase I completed
**BB128**	BiomeBank	MAA	Marketing authorization application submitted in Australia
**Ridinilazole** (Fast-track, breakthrough, and QIPD)	Summit Therapeutics	Prevents cell division	Phase III completed
**Ibezapolstat** (QIDP and fast-track)	Acurx Pharmaceuticals	Inhibitor of DNA polymerase IIIC	Phase III
**CRS3123**	Crestone Pharma	Methionyl-tRNA synthase inhibitor	Phase II
**DNV3837** (Fast track and QIDP)	Deinove	Inhibits bacterial DNA gyrase and protein synthesis	Phase II
**MGB-BP-3** (QIPD and fast-track)	MGB Biopharma	Transcription inhibitor	Phase III
**Alanyl-L-glutamine**	University of Virginia	Blocks caspase-8 activation	Phase II
**TNP-2198**	TenNor Therapeutics	RNA polymerase inhibitor	Phase Ib/IIa
**IM-01**	ImmuniMed	Neutralizes the CD toxins	Phase II
**LMN-201**	Lumen Bioscience	Neutralizes CD toxins and destroys the cell wall of CD	Phase II/III
**NTCD-M3**	Destiny Pharma	A non-toxic strain of CD that makes a “ground cover” in the colon	Phase III

**Table 3:** Drugs not in active development.

Drug	Developer	Mechanism of Action	Reference
**DS-2969b**	Daiichi Sankyo	GyrB antagonist	[75,114]
**OPS-2071**	Otsuka Pharmaceutical	DNA gyrase inhibitor	[75,115,116]
**Cadazolid**	Actelion Pharmaceuticals	Protein synthesis inhibitor	[22,75,117]
**Misoprostol**	Vanderbilt University and the National Institutes of Health	Prostaglandin analog	[92,118]
**Ramoplanin**	Nanotherapeutics	Cell wall synthesis inhibitor	[75,119,120,121]
**KB109**	Kaleido Biosciences	Microbiome metabolic therapeutic	[75,122]
**LFF571**	Novartis	Protein synthesis inhibitor	[22,123]
**Ramizol**	Boulos & Cooper Pharmaceuticals	Inhibits mechanosensitive ion channels of large conductance (MscL)	[22,124]

## Data Availability

The data mentioned in the text has been taken from the cited references.
